# HIV–AIDS Stigma in Burundi: A Qualitative Descriptive Study

**DOI:** 10.3390/ijerph18179300

**Published:** 2021-09-03

**Authors:** Néstor Njejimana, Lucía Gómez-Tatay, José Miguel Hernández-Andreu

**Affiliations:** 1 Escuela de Doctorado, Universidad Católica de Valencia San Vicente Mártir, 46001 Valencia, Spain; nesnje@mail.ucv.es; 2 Institute of Life Sciences, Universidad Católica de Valencia San Vicente Mártir, 46001 Valencia, Spain; jmiguel.hernandez@ucv.es; 3 Grupo de Medicina Molecular y Mitocondrial, Facultad de Ciencias de la Salud, Universidad Católica de Valencia San Vicente Mártir, 46001 Valencia, Spain

**Keywords:** HIV/AIDS, stigma, discrimination, Burundi, Africa, PLWHA

## Abstract

HIV/AIDS stigma is a global issue and a serious problem in African countries. Although prevalence remains high in this region, no detailed study has yet been carried out to determine and characterize this problem in Burundi. Using a qualitative analysis based on an extensive series of 114 interviews, we describe the main characteristics of HIV stigma in the country. The results of our study indicate that the problem of HIV/AIDS stigma is widespread in Burundian society, as all participants in the research reported having experienced some kind of HIV stigma. The seven dimensions of stigma identified in people living with HIV/AIDS (PLWHA) in Burundi are physical violence, verbal violence, marginalization, discrimination, self-stigma, fear and insecurity, and healthcare provider stigma. These dimensions of stigma can be experienced through different manifestations, which have been characterized in this study, revealing that the problem of stigma in PLWHA is still an important issue in Burundi.

## 1. Introduction

### 1.1. AIDS in Burundi

The first case of acquired immunodeficiency syndrome (AIDS) was reported in Burundi in 1983. Since then, the extent of the infection in the general population has been assessed through three successive national seroprevalence surveys, conducted in 1989, 2002, and 2007. The last of these surveys showed an overall seroprevalence rate of 2.97% in the Burundian population, with rates varying between urban and rural areas, being higher in urban areas (4.59% vs. 2.82%) [1].

The 2016–2017 Burundi Demographic and Health Survey provides the most up-to-date data on the epidemiological situation of human immunodeficiency virus (HIV)/AIDS in the country. According to this survey, 0.9% of men and women between the ages of 15 and 49 are HIV-positive. HIV prevalence is slightly higher among women (1.2%) than men (0.6%), and is more than three times higher in urban (2.5%) compared to rural areas (0.7%) [2].

On 26 July 2017, during her speech in the National Assembly, Dr. Nijimbere Josiane, Burundian Minister of Public Health and the Fight against AIDS, reported that, in 2017, 80,255 people were living with HIV/AIDS in Burundi, 51,917 of whom were already taking antiretroviral treatment (ART), as noted in an AGnews article on 31 July 2017. Out of an estimated population of 10,509,346 inhabitants in 2017, this suggests that 0.76% of the Burundian population is living with HIV/AIDS.

In Burundi, the fight against HIV is considered a priority, as illustrated by the implementation and development of national strategic programs in 2002–2006, 2007–2011, 2012–2016, 2014–2017, and 2018–2022 to fight AIDS. These programs seek universal access to quality HIV/AIDS prevention, treatment, and support services. Most notable was the development in 2011 of the National Program to Combat AIDS and Sexually Transmitted Infections (Programme de la Lutte Contre le SIDA et les Infections Sexuellement Transmissibles, PNLS), and the creation of the National Council of Fight against AIDS (CNLS) [3].

The current HIV situation in Burundi shows that important steps have been taken in raising awareness of the prevalence of HIV/AIDS and its diagnosis in recent years, with cases having fallen significantly from a seroprevalence in 2002 of 6% to 0.6% in 2018. Burundi has been able to apply the diagnostic policy to 80% of its population, as noted in a Voice of America article on 3 December 2018.

With the aim of definitively defeating this disease, Burundi has embraced the World Health Organization (WHO) vision of working towards a world in which there is no new HIV infection, no HIV-related deaths, no type of HIV-related discrimination, and in which people infected with the virus can live long and healthy lives. To achieve this, the WHO published the “Global health sector strategy on HIV 2016–2021” [4], a project that guides efforts to accelerate and focus initiatives to prevent virus transmission, allow more people to know their serological status, provide ART and comprehensive and long-term care to all infected people, and to help tackle stigma and discrimination.

The importance of associations that work in Burundi to help PLWHA should also be highlighted, such as the “Association Nationale de Soutien aux Séropositifs et Malades du SIDA (ANSS)” and SWAA Burundi (Spa Wellness Association of Africa in Burundi), whose objective is to promote the prevention of HIV transmission and improve the well-being of PLWHA through actions such as providing medical and psychosocial care or helping to develop economic strategies in different communities.

### 1.2. The Problem of HIV-Related Stigma in Burundi

It is well established that HIV/AIDS-related stigma is currently a serious problem among AIDS patients globally, and Africa is no exception [5]. It has implications for the health and well-being of PLWHA at many levels [6], causing psychological problems such as depression, low self-esteem [7], stress [8,9], and suicidal ideation [10,11]. Moreover, fear of discovery leads PLWHA to hide their seropositivity [12,13,14], making it difficult to treat the disease, as stigma has also been associated with non-adherence to ART [15].

The WHO, in its document ‘HIV testing, treatment and prevention: generic tools for operational research’, whose purpose is to promote AIDS research to inform effective policies and services in the fight against this disease, dedicates one of its four chapters to stigma and discrimination associated with HIV. It highlights the need to conduct research in this regard and to collect information from “different geographic, sociodemographic and cultural settings” [16]. The ultimate interest of this research is to provide evidence on the basis of which develop appropriate interventions to tackle the problem of HIV stigma in particular settings [17]. Stigma being a multi-level phenomenon, interventions can and should operate at different levels [18,19]. Strategies such as counseling or self-help operate at the intrapersonal level. Support in the person’s environment operates at the interpersonal level. At the community level, interventions focus on attitudes and behaviors and strategies include education and advocacy. Training programs and institutional policies operate at the organizational/institutional level. Finally, law enforcement and policy development are strategies to tackle stigma at the governmental/structural level [20]. There are numerous studies on the effectiveness of different interventions to reduce HIV stigma in particular contexts and with diverse target populations [21,22]. For example, at the intrapersonal level, a stress-reduction strategy was shown to reduce self-stigma and fear to disclosure in PLWH in Zimbabwe [23], while a group-based behavioral intervention (including information provision, acquisition of coping skills, and contact with youth living with HIV) was not effective for adolescents and young adults newly diagnosed with HIV [24]. Online platforms where PLWHAs can talk about HIV stigma have also proven effective at this level [25]. At the interpersonal level, interventions targeting family members of PLWHA can be effective, although these types of approaches are currently scarce [26]. At the institutional level, strategies typically focus on reducing HIV stigma at healthcare settings [27,28]. An example of a community level intervention was carried out in five churches in a high HIV prevalence area of Los Angeles County, including strategies such as HIV education, pastor-delivered sermons on HIV, and HIV testing events [29]. One example of a structural-level approach to combat HIV stigma in rural Kenya consisted of a livelihood intervention, which involved giving a loan and a training program on farming techniques and harvest handling and marketing. This intervention resulted in a decrease in both self-stigma and negative community attitudes towards PLWHA [30]. Media-based HIV programs are also an example of structural-level strategies [31].

Although several studies have addressed the issue of HIV-related stigma in the African context, or include African countries among other countries studied [32,33,34,35], this issue has not been examined in depth in Burundi.

With regard to stigma in the medical and family environment in Burundi, Dr. Biziragusenyuka Jérémie, national coordinator of the ESTHER project (Ensemble pour une Solidarité Thérapeutique et Hospitalière en Ré seau), a tool for bilateral cooperation between the governments of France and Burundi in the field of AIDS, denounced the situation of women. She reported that HIV-positive women can be expelled from their homes due to the influence of their husband’s relatives, such as mothers-in-law or brothers-in-law, for having revealed their HIV-positive status or for not having breastfed their babies to protect them against HIV, as noted in an ARIB article on 28 May 2014. Likewise, she warned that healthcare providers are also involved in the stigmatization and discrimination of patients with HIV/AIDS in Burundi in various ways, such as refusing to provide care to pregnant women with HIV during labor and childbirth or to treat newborns from HIV-positive mothers, or by making inappropriate comments and engaging in disrespectful behavior towards AIDS patients. One particular problem is that of widowed women, who are often persecuted by the relatives of their deceased husbands, who reject them and even steal property and land to force them to return to their parental home, as noted in a Touki Montréal article on 9 July 2011. In 2005, an important step was taken in recognizing the rights of PLWHA, when the government of Burundi passed a law to end discrimination against AIDS patients, “Law 1/018 of 12 May 2005 on the Legal Protection of Persons Infected with HIV and Persons with AIDS (2005)” [36], imposing fines of 10,000 to 100,000 Burundi Francs (Fbu), as well as administrative, disciplinary, and other sanctions intended to punish those who violate the law.

An extensive literature search has revealed a lack of detailed studies on the problem of HIV stigma in Burundi that include the systematic conduct of interviews and collection of information on this issue among individuals of the Burundian population. Thus, the objectives of this study were to describe the real-life experiences of HIV-positive people and reveal the scope and typology of HIV stigma in PLWHA in the Burundian region. First, we introduce a series of identified dimensions of the HIV stigma experienced by PLWHA in Burundi. Second, we quantify the extent of each of them in terms of the percentage of participants who reported having experienced at least one manifestation of each dimension of HIV stigma. Third, we characterize HIV stigma experienced by PLWHA in Burundi.

## 2. Materials and Methods

### 2.1. Conducting Interviews

In the period from 9 March to 10 April 2020, 114 PLWHA were interviewed at the Ambulatory and Multidisciplinary Care Center for People Living with HIV/AIDS (CPAMP: Centre de Prise en Charge Ambulatorie et Multidisciplinaire des Personnes Vivants avec le VIH/SIDA) in the Kamenge Hospital-University Center (CHUK: Centre Hospitalo-Universitaire De Kamenge) in Burundi. Participants’ ages ranged between 18–81 years (mean age 46.41 years, standard deviation [SD] 11.88); 85 were women and 29 were men. All participants were of legal age, which according to the legislation in force in Burundi, is 18 for women and 21 for men.

The interviewees visited the center for various reasons, such as to collect ART, request a CD4 test, or to consult with their doctor. This center was chosen because it is the only university hospital in Burundi and is attended by PLWHA from all over the country, although most come from the capital. The nurses informed the patients of the possibility of participating in the research. Those who wanted to participate were interviewed by N.N. (main researcher).

A qualitative descriptive design was adopted for this study, in order to achieve an in-depth understanding of the stigma-related experiences of the patients. Structured interviews were carried out by N.N. in the local language, Kirundi, in order to ensure optimal patient communication, and the answers were written down on paper by the researcher, who personally stored the documents. The questionnaire was developed ad hoc with the help of a psychologist, trying to cover both psychological and relational aspects.

The study was conducted in accordance with the provisions of the Declaration of Helsinki. Ethical approval for the study was granted by the Managing Director of the Centre Hospitalo-Universitaire de Kamenge (N/Ref: 2020/ACGHUK.440/11.5). All participants signed an Informed Consent, which the principal investigator also explained to them verbally, before they gave their statements.

To carry out the interviews, we developed a questionnaire consisting of eight questions, three of them related to the psychological aspects of PLWHA and five related to the dynamics of social relations between PLWHA and Burundian society.

To learn about the psychological experiences of PLWH in Burundi, the following questions were asked:

1. Can you tell us how you found out and accepted your HIV status?;

2. How do people around you respond to your HIV status?;

3. How do you feel about your friends?

Secondly, in order to understand the dynamics of the relationships between PLWHA and Burundian society, the following questions were asked:

1. How is your relationship with your children?;

2. How is your relationship with people around you?;

3. How is your relationship with your doctors and social workers?;

4. How is your relationship with your co-workers?;

5. How is your relationship with your friends?

The 114 PLWHAs interviewed answered the eight questions in the questionnaire. Their answers were then analyzed to identify and characterize the dimensions of stigma experienced by PLWHA in Burundi, and to show its extent in the Burundian population.

### 2.2. Testimonial Analysis

Given the diversity of categorizations available for the study of HIV-related stigma and the scarcity of studies that explore the experiences of PLWHA in Burundi, we decided to establish the dimensions of stigma in these patients after analyzing the interviews, thereby preventing conditioning of responses to predetermined categories.

Interviews were reviewed to identify the different dimensions of stigma experienced by our research population. The thematic analysis was carried out in various steps similar to those proposed by Braun and Clarke [37]. The texts of the 114 interviews, transcribed in Kirundi, were reviewed by N.N. to identify different manifestations of stigma associated with HIV (subthemes). The initial list of identified manifestations of stigma was refined in a process in which all authors participated, grouping those categories that were too similar. In a second review of the testimonies, it was registered which participants reported each of the stigma manifestations found (using a coding system of ones and zeros, according to the presence or absence of the manifestation of stigma in each testimony). The text fragments evidencing these manifestations were highlighted in bold. N.N. translated 43 of the interviews into Spanish, enough to include testimonies referring to all the identified manifestations. These testimonies were reviewed by the other authors to corroborate the identified manifestations, verify the adequacy of the coding system, and select the extract examples. Subsequently, the different manifestations found were grouped in different dimensions of HIV stigma (themes). In this process, the available literature on HIV stigma (Table 1) was taken into account to extract and use the terms in line with previous research as far as possible, although this did not exclude the possibility of finding new dimensions of stigma in this population [38].

## 3. Results and Discussion

### 3.1. HIV Stigma Dimensions Experienced by PLWHA in Burundi

Taking the aforementioned classifications as a reference, after analyzing the statements, we identified a series of dimensions of the HIV stigma experienced by PLWHA in Burundi (Table 2). This gives us more insight into the problem, with the ultimate aim of informing future actions to alleviate stigma effectively.

### 3.2. Extent of the Different Manifestations of Stigma in PLWHA in Burundi

**Figure 1:**
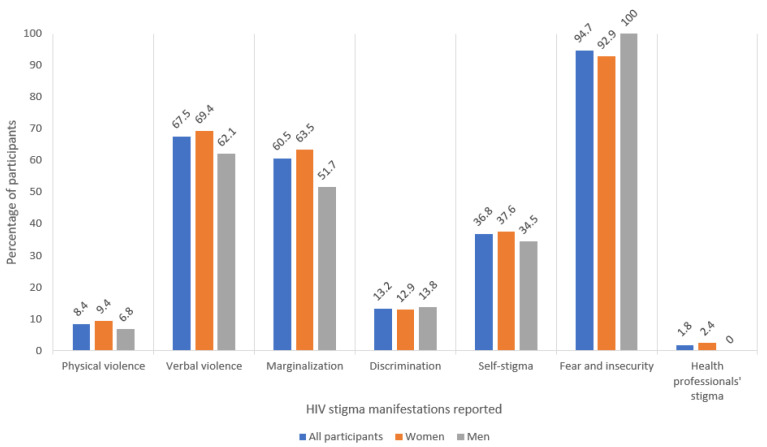
Percentage of participants who reported having experienced each HIV stigma dimension.

Once the different dimensions of stigma experienced by our research population was identified, we then quantified the extent of each of them in terms of the percentage of participants who reported having experienced at least one of the manifestations of each dimension of HIV stigma. The main results of this analysis are presented in Figure 1, showing the total percentage of all participants, women and men who have experienced each kind of stigma dimension. A more detailed breakdown of data is provided in Table 3, where the different manifestations of each HIV stigma dimension are also quantified.

### 3.3. Characterization of HIV Stigma Experienced by PLWHA in Burundi

Finally, we carried out a descriptive study of the statements, in order to characterize the different dimensions of HIV stigma experienced by PLWHA in Burundi. Below, we refer to the different manifestations of the HIV stigma dimensions identified, illustrated with extracts from the statements analyzed.

#### 3.3.1. Physical Violence

Almost one-tenth of the participants (8.4%) reported having suffered physical violence due to their PLWHA condition. Although the percentage is relatively low compared to other forms of stigma, this dimension is nevertheless very serious. In addition, the percentage is higher than that found in recent similar studies in other African countries. Thus, in Malawi, Burkina Faso, Uganda, and Kenya, the prevalence of this type of stigma ranges from 2.1% to 3.1% [41].

Interestingly, from the analysis of the interviews, we can conclude that all the cases of physical violence occurred within the couple relationship, more often from the man towards the woman, with 9.5% of the women and 6.7% of the men reporting having experienced it. Thus, participant 23, a 54-year-old woman, recounted the violent reaction of her husband upon learning that she was infected with the virus, despite the fact that he had hidden his seropositivity from his wife:

“In my immediate circle, the only person who was violent towards me was my own husband [...] he accused me with violence of causing his HIV infection and threatened me”.

We do not have any testimony that reflects concrete examples of physical violence, because, due to a cultural reason, respondents expressed this fact with discretion, in the way it is shown in the testimony provided.

#### 3.3.2. Verbal Violence

This is a very widespread dimension of stigma, with 67.5% of the participants reporting having experienced at least one of its manifestations.

Unpleasant words, insults. One third of the participants (33.3%) reported having experienced unpleasant words or insults for being PLWHA. This percentage exceeds in all cases the frequency of similar manifestations identified in Malawi, Burkina Faso, Uganda, and Kenya, namely ‘made to feel badly’ (13.8%–28%), ‘been told HIV is what I deserve’ (2.3%–21.1%), and ‘verbally abused or ridiculed’ (15%–17.8%) [41].

Participant 35 (woman, 52 years old) explained:

“Sometimes the neighbors insult us for being PLWHA, but the biggest problem comes from my husband’s family”.

Participant 40 (man, 40 years old) reported:

“…they go even further: They also tell me I don’t have much time left to live and that AIDS will kill me soon”.

Accusations. Accusations were the least frequent form of verbal violence, reported by 14% of the participants. Participant 1 (woman, 42 years old) reported:

“My husband’s family just started accusing me of being guilty of my husband’s infection. Then they started to make my life miserable”.

Participant 40 (man, 40 years old) reported:

“It hurts me a lot and makes me sad when I hear some of them openly saying that I murdered my dear wife”.

Gossip, criticism of the PLWHA behind their back. Half of the interviewees (50.9%) claimed to have been victims of gossip or criticism behind their backs.

Participant 1 reported that:

“When the neighbors found out that we [my husband and I] were HIV positive, they began to speak ill of us. […] We lead a surprisingly normal life like everyone else, but we experience a new form of subtle stigma that manifests itself in finger-pointing (“kutunga agatoki”)”.

Participant 11 (man, 47 years old) also reported a similar experience:

‘When some neighbors found out that I had HIV, they started pointing their fingers at me. They said I was going to die soon and that I was suffering from the consequences of my debauchery. But when I was present, they were kind and understanding. It was later that I found out that my neighbors are hypocrites and that they spoke ill of me behind my back’.

#### 3.3.3. Marginalization

A high percentage of those interviewed, 60.5%, reported having experienced some form of marginalization due to their PLWHA status.

Rejection by spouse. Among those who rejected the PLWHA when finding out their seropositivity, are spouses, a situation reported by 12.6% of those interviewed. In the Malawi, Burkina Faso, Uganda, and Kenya study, the percentage of participants who reported being ‘abandoned by a spouse’ ranged from 6% to 12.7% [41]. Therefore, for this HIV stigma manifestation, our data are within the range of these neighboring countries, but very close to the highest limit.

Participant 6 (woman, 50 years old) recounted how her husband divorced her when he found out her HIV positive status:

“Since I didn’t quite understand how I could be HIV-positive without being unfaithful to my husband, I asked for a test and my HIV-positive status was confirmed. […] I told my husband everything and he immediately decided to divorce me and kicked me out of his house”.

Rejection and persecution by other family members. Rejection of PLWHA on other occasions (18.4%) comes from other family members, typically the spouse’s family.

Thus, participant 35 (woman, 51 years old) stated:

“I am unjustly persecuted and considered a criminal in my husband’s family”.

Sometimes other family members are the ones who reject the PLWHA, for example, children, as in the case of participant 40 (man, 40 years old):

“Of my six children I have good relationships with only two daughters, but with my four sons, the relationships are not good. They want me to die and they openly say that it would be best for them if I died quickly so they could keep my things”.

Sometimes, the rejection in the family becomes generalized. Thus, participant 37 (man, 45 years old) gave us this testimony:

“Knowing the stigma that PLWHA have to endure in my setting, I decided to hide my seropositivity. After the decision, I thought it would be better to tell my mother, convinced that she would know how to keep my seropositivity secret, but I was wrong, because then she told the whole family, who began to avoid me little by little and now most of them refuse to meet me. […] My relatives, even those closest to me, began to distance themselves from me. I was pushed aside to the point of feeling lonely”.

Refusal to share things with them and touch the same things (plates for food, closet for clothes). This is another form of marginalization of PLWHA, experienced by 15.1% of those interviewed. Participant 1 (woman, 42 years old) recounted the marginalization that she experienced at work in this regard:

“I found out that I was HIV positive in 2005 and I told my co-workers. I was wrong because then they began to stigmatize and discriminate against me, avoiding any relationship with me. Now relations have improved and we are working closely together. But between 2005 and 2011, I suffered real stigma, some colleagues did not want to share some work tools and common places such as bathrooms with me”.

Participant 7 (woman, 28 years old) experienced this type of marginalization from her adoptive family:

“When I was still living with my adoptive family, my adoptive mother always made sure to keep my clothes separate from her own daughters’. She kept telling us very insistently that it was in our own interest to never use the same toothbrush and that each of us had to have her own nail clippers. There was then a separate closet for my clothes and another for her and the other children. […] No one used my dish or my glass”.

In other cases, it is friends who are aware of PLWHA seropositivity who take these types of unnecessary precautions, as happens to participant 35 (woman, 51 years old):

“My relationships with my friends are quite good, but their children are not allowed to eat or drink anything in my house, thinking that I might infect them. When they send their children to my house out of necessity, they always tell them not to take too long”.

Social isolation. Our analysis revealed another manifestation of marginalization associated with HIV, social isolation, experienced by 11.4% of participants. In a study in four other African countries, the percentages for the manifestation “excluded from social events” ranged from 1% to 13.9% [41]. Again, even within the considerably wide range found in these neighboring countries, Burundi is near the high end.

In our study, this manifestation of HIV stigma was generally exemplified by the exclusion of PLWHA from social events such as parties. Thus, participant 29 (woman, 43 years old) stated:

“The proof of the intolerance of my neighbors towards PLWHA is that when there are parties, for example, they refuse to invite them like everyone else”.

Abandonment in hospitals by their relatives. Even though they are exceptional cases (1.8%), due to the severity of the stigma, we can highlight the presence of two cases in which the PLWHA was abandoned in the hospital by relatives, thinking that AIDS has no cure and it is a waste of time to continue taking care of them.

Thus, participant 17 (woman, 34 years old) related her experience regarding her HIV-positive husband, when he fell into a coma and was hospitalized for 6 months:

“Members of his own family helped him in the beginning, so I could continue working. But little by little they became discouraged and left him in the hospital. As a wife, I was forced to quit my job […] to take care of him”.

Distancing by neighbors. One quarter of participants (25.4%) claimed to have become distant from their neighbors because of being HIV positive. Participant 25 (woman, 40 years old) tells in her testimony:

“When the neighbors found out that I was HIV positive, they isolated me by refusing to sit with me and share some objects with me. Now the situation is changing so that overt stigma is increasingly giving way to subtle discrimination. My neighbors continue to insult PLWHA but without anyone noticing so as to avoid a complaint that could force them to pay the fines”.

Banning of children from playing with those of HIV-positive parents. This is another form of marginalization reported by 6.1% of the participants, in which the HIV stigma is extended to the children of PLWHA.

Accordingly, participant 40 (man, 40 years old) reported:

“When my neighbors found out about my HIV status, they took it very badly. They directly started stigmatizing and discriminating against me, calling me a low life and worthless person. They have gone so far in discrimination to even prevent their children from playing with mine”.

#### 3.3.4. Discrimination

*Difficulty in getting a job.* The difficulty in finding employment due to having HIV is, within the ‘discrimination’ dimension, the least frequent manifestation of HIV stigma, reported by 3.5% of the participants.

In this sense, participant 35 (woman, 51 years old) related how it was impossible for her to advance in her job as a teacher due to her condition as PLWHA, since her boss considers her limited by her illness:

‘The principal knows my serological situation, she assumes I’m a frail teacher, although I feel very well and work more than some of my colleagues. She has refused to give me my own class and I’m always considered a helper. When there are training seminars she never sends me to participate in them, and I feel discriminated against and stigmatized’.

Similarly, participant 14 (man, 23 years old) said that he has to hide his HIV positive status at work in order not to lose his job:

‘Knowing that it is almost impossible to do domestic work being HIV positive in our society, I had to hide my HIV positive status from my boss and I never told him to avoid stigma. […] In order to hide my seropositivity, if I have an appointment to go to the hospital to pick up antiretroviral treatment, I take advantage of the time to go shopping at the market. I do everything in total discretion to avoid losing my job’.

*Difficulty in obtaining company loans at work.* A small percentage of the participants (5.3%) mentioned how the process of granting loans in companies is discriminatory towards PLWHA.

In this regard, participant 8 (woman, 48 years old) reported:

‘I realized that when our company has to grant collective loans to workers, it is difficult for PLWHA to get them compared to other workers’.

*Eviction from rental houses by owners.* The most frequent manifestation of HIV stigma in the dimension of ‘discrimination’ was eviction from rental houses due to HIV status, reported by 6.1% of the participants. In the study in four other African countries, the percentages for the category ‘expelled from place of living’ ranged from 0 to 3.9 [41], so according to our data, this problem appears to be more frequent in Burundi than in these neighboring countries.

Participant 9 (woman, 47 years old) has already been evicted twice from her rented home:

‘Twice, the rental home owners kicked me out for no apparent reason’.

#### 3.3.5. Self-Stigma

This dimension of HIV stigma has been experienced by 36.8% of participants. In comparison with the data of the study on four other African countries, where the percentages for ‘internalized’ stigma are 9.6% (Malawi), 29.3% (Uganda), 32.3% (Kenya), and 45% (Burkina Faso) [41], Burundi ranks fourth out of five in the frequency of this dimension of HIV stigma.

*Shame.* Shame was the most common manifestation of this dimension of HIV stigma: one quarter of the participants (25.4%) admitted to feeling this form of self-stigma.

Participant 42 (man, 60 years old) reported:

‘I am very ashamed when I am with my friends and it hurts me to know that I am infected with HIV and they are supposedly healthy’.

*Guilt.* The feeling of guilt is also present in the study population, constituting another of the manifestations of self-stigma in PLWHA in Burundi.

Participant 33 (woman, 57 years old) told the interviewer:

‘Thank God, when I told him, my husband did not react violently as I expected. But I felt a lot of shame and guilt as if I had infected him voluntarily’.

*Attempted suicide.* This form of self-stigma is not high (2.6% of the participants), but its seriousness means it should be taken into account when addressing the problem of HIV stigma in Burundi.

Participant 40 (man, 40 years old) recounted how being diagnosed with AIDS led him to attempt suicide:

‘My doctor sent me to have an AIDS test in a hospital and they discovered that I am HIV positive. When they told me the result, I was terrified, I felt I didn’t deserve to live and I tried to commit suicide. I believed that my death was near and I considered myself as a man without a future and I didn’t deserve to live, I considered myself already dead because there was no reason for me to hope’.

#### 3.3.6. Fear and Insecurity

Fear and insecurity constitute the most frequent dimension of HIV stigma in Burundi, according to our data, with 94.7% of the participants reporting some of its manifestations.

*Fear of illness, fear of dying soon.* Although the diagnosis of many diseases can be poorly received by patients, and many diseases can cause fear, we have considered this category as a manifestation of HIV stigma after detecting a divergence between the current prognosis that the treatments available for AIDS patients allows, and the perception by many of these patients in this regard, which remains highly distorted and negative. Half of the participants (50%) reported experiencing this manifestation of HIV stigma.

Hence, participant 23 (woman, 54 years old) recounted how when she was diagnosed as HIV positive, she thought that she had little time to live:

‘The serology test they did there revealed my HIV positive status. When the doctor told me, I momentarily lost my mind thinking that I was going to die soon. When I got home, I sadly told my children that I could die soon and they all began to cry’.

*Fear that their HIV status will be discovered.* This manifestation of stigma was found to be highly prevalent among PLWHA in Burundi. Some participants feared that they would be rejected by family, friends, or acquaintances if their HIV status was discovered.

Accordingly, participant 13 (woman, 46 years old) reported:

‘The people around me did not find out about our HIV positive status. We decided to keep it a secret to avoid stigma and discrimination. We have seen here and there how our neighbors have little tolerance for PLWHA who have been careless enough to tell people. [...] Although they don’t know anything, I’m afraid that one day they will find out and start stigmatizing us. The only people who know that I am PLWHA are my brothers and sisters, my own mother and one of my children. Discretion is essential to escape the stigma of the environment’.

Added to this fear is the inconvenience of having to hide their status in their social relationships. Along these lines, participant 6 (woman, 50 years old) gave the following testimony:

‘When I am with my friends, I feel uncomfortable because sometimes they ask me the reason for my divorce, and I feel ashamed because I cannot tell them the whole truth for fear of being discriminated against. The only people who know the truth about my divorce are my two sisters and we have agreed to keep it a secret so as to avoid stigma from my friends.’ She also commented: ‘The relationships I have with my co-workers are normal because they don’t know that I am HIV positive. In our conversations, I always avoid talking about it to avoid the problem of stigma’.

Many participants stated that they cannot collect and take their ART openly for fear of their HIV status being discovered. Thus, participant 7 (woman, 28 years old) reported:

‘I refused to go to boarding schools for fear that my classmates would ask me why I always take the pills when my health is apparently normal’.

For the same reason, participant 33 (woman, 57 years old) collects her ART in a center far from her home, to avoid meeting acquaintances:

‘I always prevent acquaintances from seeing me pick up the medications. It is precisely for this reason that I come to receive antiretroviral treatment at the Roi Khaled hospital in Kamege instead of picking it up in the Ngagara district closer to my home’.

#### 3.3.7. Health Professionals’ Stigma

All the PLWHA interviewed say they have good relationships with their current healthcare team. Only two participants mentioned having suffered this type of stigma in the past, as reported below.

Participant 13 (woman, 46 years old):

‘The current healthcare system is very welcoming to us. But not long ago, there was a lady in this hospital who I do not want to mention because she did not treat us well. The current healthcare team is very good and I have a very good relationship with all the team members’.

Participant 29 (woman, 43 years old):

‘The healthcare team welcomes us very warmly and some have become my friends. For example, doctor […] has become a great friend of mine. I trust him completely and that is why I’m not afraid to tell him anything that concerns me about my health. Those who told us off are no longer on this current team’.

These were the only cases in our study in which this type of manifestation of HIV-related stigma was reported. Although some studies show that stigma in the healthcare setting was greater in the past, or report how healthcare providers are an important support for PLWHA, other studies have shown that this form of stigma remains, comprising behaviors such as fear of contact, delay of services, substandard services, refusal of care, rudeness of healthcare providers, breach of confidentiality, and poor patient follow-up [32]. The fact that the interviews were carried out in the hospital and that all the interviewees were treated by the same doctors is a limitation of this study when describing health professionals’ stigma in Burundi, since it could restrict the freedom of the participants to express negative opinions about the staff who treat them, or that other healthcare teams could be responsible for this type of stigmatization.

## 4. Conclusions

Analyzing the different testimonies obtained through the interviews, we can affirm that the stigma of PLWHA is a current reality in Burundi, since all the PLWHA interviewed reported having experienced at least one of the dimensions of this stigma. Only four of the 114 interviewees reported having experienced only one of the identified manifestations of HIV stigma, with the majority having experienced several. The seven dimensions of HIV stigma identified in PLWHA in Burundi are physical violence, verbal violence, marginalization, discrimination, self-stigma, fear and insecurity, and health professionals’ stigma. These dimensions of the stigma can be experienced through different manifestations, which have been described in this paper.

In terms of the frequency of the different dimensions and manifestations of HIV stigma, no significant differences were found between men and women. The percentages found in our study were higher than those found in other African countries for the manifestations of HIV stigma in the categories of ‘physical violence’, ‘unpleasant words’, ‘insults’ (verbal violence), and ‘eviction from rental houses by owners’ (discrimination). Despite this, some of the interviewees reported that these forms of open stigma are decreasing, thanks to legislation penalizing them. Nevertheless, it has not disappeared entirely, but is still very present in its subtle form, ‘criticism behind the back’ and different forms of ‘marginalization’, as corroborated by our analysis of the statements of participants (50.9% and 60.5%, respectively). According to our data, ‘fear and insecurity’ is the most common HIV stigma dimension in PLWHA in Burundi (94.7%), especially ‘fear that their HIV status will be discovered’, which is a concern for 89.5% of these patients. Particularly serious manifestations, such as physical violence and suicide attempts, have low but significant frequencies (8.4% and 2.6%, respectively). Interestingly, all of the reported cases of physical violence occurred within the couple relationship, most often from the man towards the woman. Discrimination appears in a considerable percentage of participants (13.2%), and is characterized in two aspects, namely labor discrimination and the unjustified eviction of PLWHA from rental houses.

Finally, although significant progress has apparently been made in eliminating stigma in Burundi, our study suggests that the fight against HIV stigma in this country is far from over. We hope the detailed characterization of this problem carried out in our study will allow the development and optimization of strategies at the national level that will help to alleviate and ultimately eradicate stigma in PLWHA in Burundi and other countries. Subsequent studies should delve into the possible variation of stigma in different regions of the country (for example, between rural and urban areas), between different medical centers (regarding health professional’s stigma) or in different population groups (women, adolescents, sex workers, etc.), with the aim of informing more specifically the development of stigma reduction interventions. As the interviewees explained, the policies developed in the country are effectively contributing to reduce open stigma (such as insults, physical violence or discrimination), but subtle stigma (such as the different forms of marginalization described) is still very present. For this reason, interventions at the interpersonal and community level should be promoted. Likewise, at a structural level it would be very useful to use the media to influence the collective awareness on this issue. Finally, our study strongly suggests that self-stigma and, above all, fear, have a high prevalence, so intrapersonal approaches continue to be essential.

## Figures and Tables

**Table 1:** Different proposals for thematic classification of HIV stigma dimensions.

Main Dimensions of HIV Stigma	Reference Where the Categorization Appears
*Social rejection*, *financial insecurity*, *internalized shame*, and *social interaction.*	[39]
*Social stigma* (prejudice which, in many cases, results in the social exclusion of PLWHA), *self-stigma* (feelings of guilt and embarrassment, of being useless, not respectable, and undesirable to other people), and *health professionals’ stigma* (related to the attitudes of these professionals towards PLWHA).	[40]
*Interpersonal discrimination* (verbal or physical abuse, exclusion, and loss of employment or housing), *discrimination in healthcare facilities,* and *internalized stigma* (feeling worthless or guilty about HIV status).	[41]
*Enacted stigma* or *stigmatizing attitudes* (prejudice and discriminatory attitudes towards PLWHA), *anticipated* or *anticipatory stigma* (expectation of PLWHA that they will experience prejudice and discrimination from others in the future), and *internalized stigma* (negative feelings of PLWHA about themselves on the basis of suffering from HIV/AIDS).	[6,34,42,43]
*Enacted stigma* (discriminatory behaviors from others), *felt stigma* (internalization of stigma), *marginalization* (other forms of social devaluation), *disclosure* (disclosure of HIV status), *morals and values*, and *visible health* (visible symptoms of AIDS status).	[44]
*Personalized stigma* (consequences that plwha perceives of other people knowing that they have HIV)*, disclosure concerns, negative self-image*, and *concern with public attitudes toward PLWHA.*	[45]
*Fear of casual transmission and refusal of contact, negative judgments about people living with hiv, internalized stigma or self-stigma, enacted stigma* (including violence, marginalization, and discrimination), *discrimination in institutional settings, discriminatory laws and policies*, and *compounded or ‘layered’ stigma* (‘HIV-related stigma that mutually reinforces and legitimates pre-existing stigma and discrimination against marginalized groups such as sex workers, injecting drug users or men who have sex with men’).	[16]

**Table 2:** Dimensions of HIV stigma experienced by PLWHA in Burundi.

1. Physical Violence
**2. Verbal Violence** *2.1. Unpleasant words, insults* *2.2. Accusations* *2.3. Gossip, criticism of the PLWHA behind their back*
**3. Marginalization** *3.1. Rejection by spouse* *3.2. Rejection and persecution by other family members* *3.3. Refusal to share things with them and touch the same things (plates for food, closet for clothes)* *3.4. SOCIAL isolation* *3.5. abandonment in hospitals by their relatives* *3.6. Distancing by neighbors* *3.7. Banning of children from playing with those of hiv-positive parents*
**4. Discrimination** *4.1. Difficulty in getting a job* *4.2. Difficulty in obtaining company loans at work* *4.3. Eviction from rental houses by owners*
**5. Self-Stigma** *5.1. Shame* *5.2. Guilt* *5.3. Attempted suicide*
**6. Fear and Insecurity** *6.1. Fear of illness, fear of dying soon* *6.2. Fear that their HIV status will be discovered*
**7. Health Professionals’ Stigma**

**Table 3:** Percentage of participants who reported having experienced different. manifestations of HIV stigma.

Hiv Stigma Dimensions and Manifestations	Total No. of Participants Who Report Having Experienced Manifestation of the Stigma (%)	Total No. of Women Who Report Having Experienced Manifestation of the Stigma (%)	Total No. of Men Who Report Having Experienced Manifestation of the Stigma (%)
1. Physical Violence	8.8	9.4	6.8
2. Verbal Violence *2.1. Unpleasant words, insults* *2.2. Accusations* *2.3. Gossip, criticism of the plwha behind their back*	67.5 *33.3* *14* *50.9*	69.4 *32.9* *12.9* *52.9*	62.1 *34.5* *17.2* *44.8*
3. Marginalization *3.1. Rejection by spouse* *3.2. Rejection and persecution by other family members* *3.3. Refusal to share things with them and touch the same things (plates for food, closet for clothes)* *3.4. Social isolation* *3.5. Abandonment in hospitals by their relatives* *3.6. Distancing by neighbors* *3.7. Banning of children from playing with those of hiv-positive parents*	60.5 *12.3* *18.4* *14.9* *11.4* *1.8* *25.4* *6.1*	63.5 *14.1* *21.2* *16.5* *11.8* *2.4* *24.7* *5.9*	51.7 *6.9* *10.3* *10.3* *10.3* *0* *27.6* *6.9*
4. Discrimination *4.1. Difficulty in getting a job* *4.2. Difficulty in obtaining company loans at work* *4.3. Eviction from rental houses by owners*	13.2 *3.5* *5.3* *6.1*	12.9 *2.4* *4.7* *7.1*	13.8 *6.9* *6.9* *3.5*
5. Self-Stigma *5.1. Shame* *5.2. Guilt* *5.3. Suicide attempt*	36.8 *25.4* *17.5* *2.6*	37.6 *29.4* *15.3* *2.4*	34.5 *13.8* *24.1* *3.4*
6. Fear and insecurity *6.1. Fear of illness, fear of dying soon* *6.2. Fear that their hiv status will be discovered*	94.7 *50* *89.5*	92.9 *50.6* *89.3*	100 *48.3* *86.2*
7. Health Professionals’ Stigma	1.8	2.4	0

## Data Availability

Data supporting reported results can be consulted by contacting the corresponding author.
